# Assessment of Occupational Exposure to Indium Dust for Indium-Tin-Oxide Manufacturing Workers

**DOI:** 10.3390/biom11030419

**Published:** 2021-03-12

**Authors:** Boo Wook Kim, Wonseok Cha, Sungwon Choi, Jungah Shin, Byung-Soon Choi, Miyeon Kim

**Affiliations:** Institute of Occupation and Environment, Korea Workers’ Compensation and Welfare Service, Incheon 21417, Korea; aromaticwind@kcomwel.or.kr (W.C.); s84one@naver.com (S.C.); ing_825@naver.com (J.S.); cbsoon@chol.com (B.-S.C.)

**Keywords:** indium, nanoparticle, ITO, indium lung disease, exposure assessment, occupational exposure, occupational exposure limit

## Abstract

According to recent research, indium nanoparticles (NPs) are more toxic than micro-sized particles. While cases of indium lung disease have been reported worldwide, very little research has been conducted on the occupational exposure to indium NPs. Recently, an indium-related lung disease was reported in Korea, a global powerhouse for display manufacturing. In this study, we conducted an assessment ofoccupational exposure at an indium tin oxide (ITO) powder manufacturing plant, where the first case of indium lung disease in Korea occurred. Airborne dustwas obtained from a worker’s breathing zone, and area sampling in the workplace environment was conducted using real-time monitoring devices. Personal samples were analyzed for the indium concentrations in total dust, respirable dust fraction, and NPs using personal NPs respiratory deposition samplers. The total indium concentration of the personal samples was lower than the threshold limit value recommended by the American Conference of Governmental Industrial Hygienists (ACGIH TLV), which was set as occupational exposure limit (OEL). However, the respirable indium concentration exceeded the recently set ACGIH TLV for the respirable fraction of indium dust. The concentration of indium NPs ranged between 0.003 and 0.010 × 10^−2^ mg/m^3^, accounting for only 0.4% of the total and 2.7% of the respirable indium particles. This was attributed to the aggregating of NPs at the µm sub-level. Given the extremely low fraction of indium NPs in the total and respirable dust, the current OEL values, set as the total and respirable indium concentrations, do not holistically represent the occupational exposure to indium NPs or prevent health hazards. Therefore, it is necessary to set separate OEL values for indium NPs. This study covers only the process of handling ITO powder. Therefore, follow-up studies need to be conducted on other ITO sputtering target polishing and milling processes, which typically generate more airborne NPs, to further investigate the effects of indium on workers and facilitate the necessary implementation of indium-reducing technologies.

## 1. Introduction

Indium, a relatively rare metal element, was used in several alloys until the use of indium oxide (IO) and indium tin oxide (ITO) began to increase in the mid-1990s with the development of flat panel display (FPD) industries, including liquid crystal displays, touch screens, and organic light-emitting devices. The increasing use of indium in these industries increased the number of workers exposed to IO and ITO aerosols. As an FPD manufacturing powerhouse, together with Japan, China, and Taiwan, Korea houses all related ITO industries, such as ITO target manufacturing, display manufacturing using ITO targets, and indium recycling companies. In all ITO-related workplaces, workers can be exposed to indium dust. Inhalation exposure to indium is known to cause indium lung diseases, such as interstitial lung disease (ILD) and pulmonary alveolar proteinosis (PAP).

In 2001, a Japanese worker engaged in the polishing process at an ITO target manufacturing plant and succumbed to an ILD-related illness, bilateral pneumothorax. This incident prompted a comprehensive reported of 10 lung disease clinical cases in indium workers from Japan, China, and the United States. Seven ILD cases and three PAP cases were recorded. When categorized by industry, eight cases occurred in target manufacturing plants, and one each in indium recovery and display manufacturing plants [1]. Interestingly, all three PAP cases were diagnosed after a relatively short working period or short indium exposure period (≤2 years). The remaining seven clinical ILD cases occurred at least three years after the first exposure. Three PAP cases were recorded in workers involved in strong physical impacts to the ITO target, such as target milling (recycling process), target polishing (manufacturing), and target blasting (finished product (LCD) manufacturing industry), during which ultrafine ITO nanoparticles (NPs) are generated.

In Korea, the OEL for indium exposure in dust was limited to 0.1 mg/m^3^, when sampled as total dust in 2016 after a series of indium-related lung disease cases were reported in Japan and the United States. This initial OEL was superseded by a stricter limit of 0.01 mg/m^3^ as the respirable dust fraction [2] in 2018. The Japanese standard is the same as the current Korean standard, and the threshold limit value recommended by the American Conference of Governmental Industrial Hygienists (ACGIH TLV) is stricter and limited to 0.0001 mg/m^3^ as the respirable indium (respirable fraction of indium dust) (as of 2019).

On the other hand, the oxidation of an element can greatly influence its toxicity to living organisms, as has been shown in previous studies on the toxicity of indium oxide particles [3,4,5]. In addition to causing lung diseases, a recent toxicity study on indium NPs determined that they exhibit a higher toxicity than nickel NPs and silica, which have been established as toxic materials [6]. Unlike microparticles, indium NPs can cause PAP at very low concentrations, necessitating the need to set separate OEL values for indium NPs [7]. Despite the hazards associated with indium exposure, few studies have investigated the level of occupational exposure to indium NPs, although several have investigated the indium concentrations of total dust and its respirable fraction.

In the meantime, a fact-finding survey was conducted for the first time in Korea from 2010 to 2011 to determine the number of cases of indium-related lung disease. Since then, there has been a gradual improvement in the workplace environment to reduce the level of occupational exposure to indium.

In Korea, the only study on indium lung disease was an epidemiologic study conducted in 2012, which reported that chest high-resolution computed tomography (HRCT) revealed that pulmonary interstitial lesions were observed in the group with high–serum indium concentrations [8]. There has been no official report on the incidences of indium lung disease despite a recent case of suspected indium lung disease reported in a worker exposed to indium powder.

The purpose of this study was to evaluate the level of indium exposure of workers and the characteristics of indium particles as part of an epidemiological investigation to determine whether the ILD case that occurred in a Korean workplace where workers handle indium oxide powder is an indium lung disease caused by exposure to indium-containing particles.

In the discussion section, we have also reviewed the patient’s serum indium level and discussed workplace indium oxide exposure and its correlation with indium lung disease.

## 2. Materials and Methods

### 2.1. ITO Manufacturing Process

Plant A manufactures an ITO sputtering target. The ITO target manufacturing process follows the following steps: ITO powder mixing, drying, pressing, sintering, machining, bonding, inspection, and shipping.

Worker A (the patient) worked in the process of collecting the mixed and dried ITO powder for 10 years. For the first six years, Worker A weighed the ITO powder, transferred it to a mixer, and collected the dried powder in a plastic container. The remaining four years were divided between continuing the same work and performing management work on the PC installed in the corridor of the manufacturing workshop, using half of the working hours for each. Worker A was diagnosed with ILD in 2017 (58 years old at the time of diagnosis).

**Figure 1:**
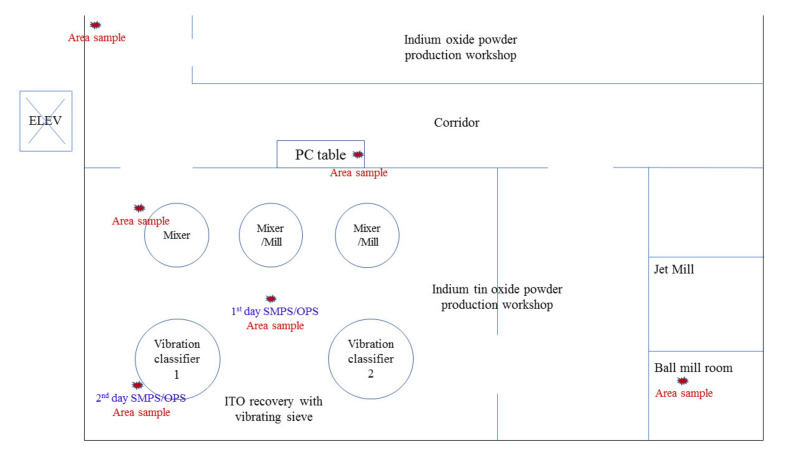
Facility layout of the indium tin oxide powder production workshop. ☆ indicates area sample measurement points.

The workshop for the ITO powder mixing and drying process is located on the third floor of the plant. Half the third floor is used for ITO powder manufacturing, and the other half for IO powder (In_2_O_3_) manufacturing from indium ingots. Worker A did not work in the IO manufacturing process (Figure 1).

The mixing operation is composed of weighing IO and tin oxide (SnO_2_) at a ratio of 9 to 1 and pouring the mixture into a mixing container with water. The mixture then passes through the container, which automatically disperses and pulverizes it, and a spray dryer, and is collected as a dried powder. The collected powder passes through the vibration classifier to bring the powder to the optimum size, and the powder product is then placed into a plastic container. When the container is full, the worker replaces it with a new one. During this process, ITO dust is released from and scattered around the vibration classifier.

After the pressing process, which is the next step, the ITO powder lumps found to be defective for the sintering process are placed into a ball mill with water and reintroduced into the mixer after being milled for several hours. The ball mill is run weekly.

Workers can be exposed to airborne indium dust when weighing IO powder on a scale, collecting the ITO powder from the vibration classifier 2–3 times daily, replacing the vinyl container, pouring the ITO lumps into the ball mill, and inspecting various facilities in the workshop.

Although the PC workbench was located in the corridor, it was not far from the vibration classifier (straight line distance: 10 m), and the entrance door of the manufacturing workshop remained open. Therefore, indium dust generated in the manufacturing workshop could affect the air of the corridor. The daily production of ITO powder is approximately 240 kg (three 80 kg containers). However, on the third day of measurement, the worker changed the container only twice.

### 2.2. Measurement and Analysis Methods

Sampling (exposure measurement) was performed three times from January to April 2019. Each sampling lasted eight hours, the workers’ daily working hours. Measurement samples were collected by personal sampling and area sampling. Personal sampling involved placing a filter in the worker’s breathing zone. Area sampling detected indium dust with a sampler fixed at a height of 1–2 m from the machine from which indium dust was released. The area sample for the classifier was placed approximately 3 m away in the first measurement and within 1 m in the second measurement.

The first measurement focused on collecting the total dust and NPs, and the same measurements were made in the second measurement on a day when the ball mill was operated. The third measurement focused on the total dust and its respirable fraction (50% of the particles, with an aerodynamic diameter of 4 µm).

The total dust and respirable dustwere sampled according to the NIOSH 0500 [9] and 0600 [10] methods, respectively. They were collected concurrently with a GilAir Plus personal pump and dual port manifold. The total dust was collected at a flow rate of 1.5 L per minute (lpm) using a 3-piece cassette mounted with a PVC (polyvinyl chloride) filter with a diameter of 37 mm and a pore size of 5 μm. The respirable dust was collected at a flow rate of 2.5 lpm using an aluminum cyclone (SKC Cat No.225-01-02) mounted with a PVC filter. The filter was subjected to a gravimetric analysis with an electronic balance after storing it for 24 h in a desiccator to remove moisture before and after each measurement.

The NPs were sampled using a nanoparticle respiratory deposition (NRD) sampler (ZNRD001, Zefon Inc., Ocala, FL, USA) mounted with a nylon filter. The particle collection efficiency curve during the diffusion stage of the NRD sampler indicated that the collected particles were smaller than 300 nm at a 50% cut-off point of 30 nm in accordance with the nanoparticulate matter deposition curve based on deposition models developed by the International Commission on Radiological Protection [11].

The PVC filter with completed gravimetric analysis of the total dust and its respirable fraction and nylon filter were analyzed for indium concentration using inductively coupled plasma mass spectrometry (ICP-MS, model Elan DRC II, Perkin Elmer Inc., Norwalk, CT, USA) in accordance with the NIOSH 7303 method [12] (given the extremely low mass of NPs, they were not subjected to gravimetric analysis).

The samples were analyzed using the in-house facilities of the Institute for Occupational Environment. The analysis laboratory has participated in every Proficiency Analytical Testing Program organized by the American Industrial Hygiene Association. The detection limit for indium analysis was set to 0.003 × 10^−3^ mg/sample.

### 2.3. Real-Time Aerosol Monitoring

Particle sizes and concentrations were measured at intervals of one min using a scanning mobility particle sizer (SMPS, model 3910, TSI Inc., Shoreview, MN, USA), which measures the particle size distribution (PSD) and particle number concentration (PNC) in the range of 0.01 to 0.38 µm, and an optical particle sizer (OPS, model 3330, TSI Inc., Shoreview, MN, USA), which measures particles in the 0.30 to 10 µm range. Each measurement sample was placed between the vibration classifier and the mixer, approximately 3 m away from the classifier on the first day, and approximately 1 m away on the third day (6 h), followed by monitoring on the PC table in the corridor (30 min) and another 30-min monitoring outside the building to obtain the control values. The measurement intervals for the SMPS and OPSwere 1 min, and theparticle densities were set to 1.2 g/cm^3^ in all the devices. The SMPS and OPS were calibrated by the manufacturer yearly to ensure the reliability and accuracy of the measured values. Real-time personal sampling was also performed on two workers (one manufacturing worker and one managing worker) during the first measurement. A personal aerosol monitor (Model SidePak AM520, TSI Inc., Shoreview, Minnesota, USA) connected to a cyclone was used to measure the real-time respirable dust concentration inhaled by the workers at one-minute intervals. In the third measurement, the lung deposited surface area (LDSA) concentration was measured every minute on the manufacturing worker using a miniature nanoparticle detector (Partector, Naneos particle solutions GmbH, Windisch, Switzerland) (instead of SidePak) to measure the LDSA concentration of the particles in the 0.01 to 10 µm range.

### 2.4. Transmission Electron Microscopy (TEM) with Energy Dispersive Spectroscopy (EDS)

Airborne particles were collected in the worker’s breathing zone for one min at a flow rate of 1 lpm using a MPS (Mini-Particle Sampler, Ecomesure, Janvry, France) [13] mounted with a TEM grid. The particle size, morphology, and chemical composition were analyzed using TEM-EDS (Model H-7650, Hitachi High Technologies, Tokyo, Japan).

### 2.5. Statistics

A distribution of the SMPS and the OPS measurement results on a log probability plot resulted in linearity. Thus, the geometric mean (GM) concentration and the geometric standard deviation (GSD) were calculated after the logarithmic conversion. ANOVA and the Scheffé’s post-hoc test were performed to compare the concentrations of PNC measured by SMPS and OPS at each measurement point. ANOVA analysis was examined at a 5% significance level, and the statistical power was 1.00 as a result of the power analysis with a large number of samples. SPSS 23.0 software (IBM Corp., Armonk, NY, USA) was used for statistical analysis, and figures were prepared using Origin 2016 software (OriginLab Co., Northampton, MA, USA).

## 3. Results

### 3.1. Indium Concentrations in Personal Samples

The total indium concentrations in the personal samples of the ITO powder manufacturing workers were 0.0684, 0.0402, and 0.0136 mg/m^3^, lower than the ACGIH TLV for the indium concentration of total dust (0.1 mg/m^3^). However, the respirable indium concentration was 0.093 × 10^−2^ mg/m^3^, nine-fold the ACGIH TLV for the indium concentration of respirable dust (0.0001 mg/m^3^). The indium concentrations of the NPs were 0.008 × 10^−2^ and 0.010 × 10^−2^ mg/m^3^, respectively.

The personal indium concentration of total dust in the managing worker was 0.0085 mg/m^3^, lower than that of the manufacturing worker, as was the concentration of indium NPs (0.003 × 10^−2^ mg/m^3^), approximately one-third of the level of the manufacturing worker. The total indium dust concentrations of the managing worker, who multitasks (manufacturing and management) in the IO and ITO processes, was also low (0.0060 mg/m^3^). However, the respirable fraction of 0.166 × 10^−2^ mg/m^3^ as indium exceeded therespirable ACGIH TLV (Table 1).

### 3.2. Indium Concentrations in Area Samples

The total dust concentration of the area samples taken on the first measurement date was the highest at the PC table placed in the corridor (0.0853 mg/m^3^), followed by the vibration classifier, mixer, and ball mill. Moreover, the respirable dust concentration was the highest at the classifier (0.0256 mg/m^3^).

The total indium concentration on the first measurement date was the highest at the ball mill (1.770 × 10^−2^ mg/m^3^), followed by the mixer (1.010 × 10^−2^ mg/m^3^), vibration classifier (0.870 × 10^−2^ mg/m^3^), and PC table (0.160 × 10^−2^ mg/m^3^). However, indium NPs were not detected around the ball mill on either measurement day (first day without and second day with ball mill operation). The highest value was detected at the PC table (0.001 × 10^−2^ mg/m^3^), followed by the mixer (0.003 × 10^−2^ mg/m^3^) and the classifier (0.004 × 10^−2^ mg/m^3^) (Table 2).

### 3.3. Total Indium, Respirable Indium, and Indium Nanoparticle Fractions

The respirable indium fraction in the total indium was 26% on average, the nanoparticle fraction in the total indium was as low as 0.4%, and the nanoparticle fraction in the respirable indium was 2.7%.

### 3.4. Particle Size Distribution Profile and Number Concentration by Sampling Location

**Figure 2:**
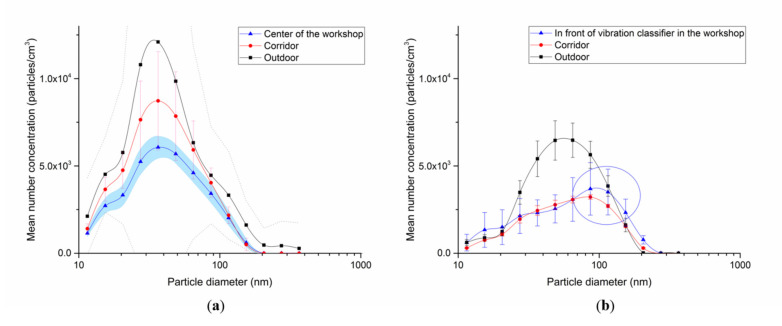
Mean particle size distribution measured bythe scanning mobility particle sizer (SMPS) on the (**a**) first and (**b**) third days, according to measurement points. The error bars, fill areas under curve, and dotted lines were standard deviations (SD). The SD of the outdoor number concentration on the first day is very large, and thus, is partially cut off in the figure.

The PNC measured using SMPS and OPS of the air was statistically significantly different depending on the sampling position (Table 3). Figure 2 shows the mean PSD and PNC measured using SMPS in the workshop on the first and third days, at the PC table in the corridor, and outside the building as the controls.

On the first day, the PSD at the three measurement points were similar, with the mode diameter measured at 37 to 50 nm. The GM of PNC was the highest outside the building, followed by the corridor and the workshop. The mean workshop PNC was 34,629 pt/cc.

On the third day, the outdoor PNC was the highest, and the mean PNC inside the workshop was 23,148 pt/cc. However, the mode diameter inside the workshop was as large as 80 nm on the third day, in contrast to that measured on the first day, presumably due to the fact that the third day measurement was performed in the immediate vicinity of the classifier and was thus affected by the indium particles discharged from the classifier.

**Figure 3:**
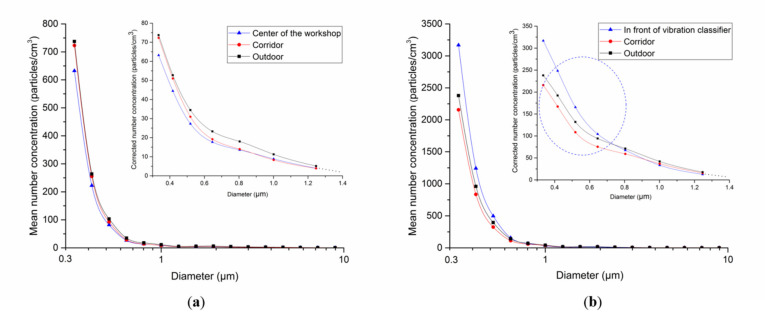
Mean particle size distribution measured by OPS in the first and third days according to measurement points. (**a**) First day, (**b**) third day; the seven size-channels covering the particle sizes of 0.33–1.25 µm are zoomed to enhance the graph readability after dividing the 0.33, 0.42, 0.52, 0.65 and 0.47 µm particle number concentration (PNC) size-channels (y axis) by 10, 5, 3, 1.5 and 1, respectively (inset).

Figure 3 displays the mean PSD calculated based on the mean PNC values measured in the workshop, corridor, and outdoors on the first and third days using OPS. Similar to the SMPS-based PNC of ultrafine particles on the first day, the PNC was the highest outdoors, followed by the corridor and the workshop, without any significant location-dependent differences (no statistical difference outdoor and corridor) (Table 3). However, unlike the SMPS result, the mean PNC value on the third day was higher in the workshop (in front of the classifier) than outdoors, and the PNC was higher in the workshop only in the PSD ranges of 0.33–0.65 µm.

### 3.5. Measurement Results of Direct-Reading Personal Samples (SidePak, Partector)

On the first day, we measured the real-time personal exposures of both workers to the respirable dust using SidePak monitor. Their mean personal exposures to respirable dust were negligibly different at 0.031 mg/m^3^ for the manufacturing worker and 0.029 mg/m^3^ for the managing worker. When the powder collection container of the classifier was changed at 11:34 a.m., the respirable dust concentration increased by approximately 60% from 0.027 mg/m^3^ (11:33 a.m.) to 0.046 mg/m^3^. During the container exchange in the afternoon, the respirable dust concentration also increased by approximately 70% from 0.027 to 0.046 mg/m^3^. However, the temporary concentration surge rapidly decreased to the background concentration due to the short exchange time of 1–2 min. On the third day, the mean LDSA of the miniature nanoparticle detector was 51.5 µm^2^/cm^3^. Moreover, a concentration increase at 09:47 am. was observed during the container exchange or inspection at the classifier, peaking at 82.1 µm^2^/cm^3^.

### 3.6. TEM Results

**Figure 4:**
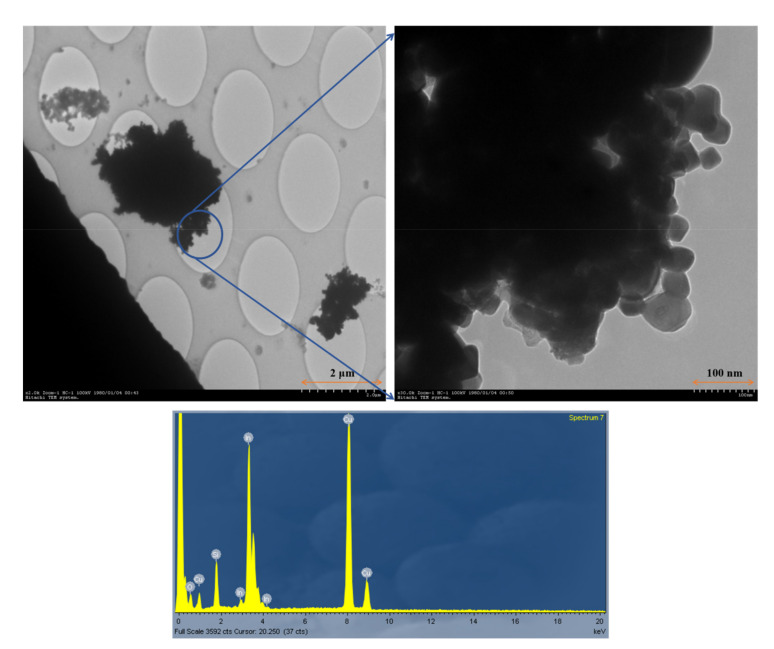
Transmission electron micrograph (TEM) of indium-containing particles and energy dispersive spectroscopy (EDS) analysis. Cu is derived from the Cu-200 mesh grid used to support the presence of particles, and silicon and oxide may be derived from the atmosphere particles.

The TEM images of the particles collected on the TEM grid showed particles with aggregated primary particles in the size range of 20–50 nm. The size of the aggregated particles ranged between 500 nm and 3 μm. Figure 4 shows the results of the chemical composition analysis of the collected particles by EDS, indicating the indium peaks.

## 4. Discussion

### 4.1. Levels of Exposure to Indium and Indium Nanoparticles

The indium exposure levels of workers manufacturing ITO powder, which were measured three times over a period of four months (January to April 2019) in this study, ranged from 0.0136–0.0684 mg/m^3^ for total indium dust, and 0.093–0.166 × 10^−2^ mg/m^3^ for respirable indium dust, which are within the range of Korean OEL (0.01 mg indium/m^3^ respirable dust). The ACGIH TLV sets separate indium standards for total and respirable dust concentrations. If the total dust standard (0.1 mg indium mg/m^3^) was applied, the exposure levels were within the recommended range. However, applying the recently set respirable dust standard (0.0001 mg indium/m^3^) revealed exposure levels exceeding the recommended maximum value.

The indium concentration of the personal sample was the highest on the second day of measurement, on which the ball mill was operated. It shows that the worker was exposed to indium when working on a ball mill.

The indium concentration in TD shows a large difference between the first and third days of measurementbecause on the third day, the ITO container was replaced fewer times than usual, and when the third measurement day visited the factory, the cleaning condition of the settled dust on the workplace floor and equipment was much better.

Although no dust is released after the ball mill operation because of the humid state, the process of placing ITO powder/cake into the mill diffuses particles into the air. In this study, the mean respirable indium fraction of the total dust was 30%, which is relatively high. A large-scale study in Korea previously estimated the fraction of respirable indium dust (*n* = 13) from the total dust (*n* = 16) at 39% [14]. A comparable Japanese study revealed the mean respirable fraction in the indium manufacturing process (*n* = 17) to be 38% (Total In/Respirable In 2.64) [15], and a similar study in the US revealed a mean respirable indium fraction of 30% (respirable In: 10.9 µg/m^3^; inhalable In: 36.5 µg/m^3^) [16], consistent with the results of this study. In contrast, in the indium-recycling plant, the proportion of respiratory indium dust was low [17].

The concentration of outdoor NPs by SMPS was higher than that in the workplace, because the atmospheric NP concentration is high in Korea due to smog in winter and spring. According to AirKorea (real-time ambient air quality monitoring system), which performed automatic measurements in the atmosphere in the vicinity of the plant, the concentration of PM2.5 on the first and third daysof measurement were 29 and 17 µg/m^3^, respectively, all of which exceeded WHO’s annual PM2.5 guidelines.

Indium related lung diseases caused by inhalation exposure to airborne indium dust primarily occur in the alveoli or a deep part of the lung [1,18]. Therefore, indium dust in an ITO related workplace is generally considered as respirable dust fraction with a mass median aerodynamic diameter of 4 μm or less, which is the size can reach the alveoli when inhaled [18]. However, according to recent toxicity research, the toxicity of indium NPs significantly exceeds that of micro-sized indium particles, and more specifically, indium NPs cause PAP, etc.

In this study, the concentration levels of workers’ personal exposure to indium NPs were measured and analyzed using NRD samplers. The workers’ personal exposure to indium NPs ranged between 0.003 and 0.010 × 10^−2^ mg/m^3^, and there is currently no global OEL for indium NPs. The NPs fraction in the respirable dust was 2.7%, considerably lower than the respirable fraction of the total dust.

TEM images revealed a size range of individual NPs between 20 and 50 nm. However, most particles formed aggregates and existed predominately in the sub-micrometer size. Therefore, the PSD profile also confirmed that the particle concentration increased predominately in the 0.33 to 0.65 mm range. This was attributed to ITO powder being weighed or placed in a container in this workshop. Thus, even if individual particles existed as NPs, they aggregated when diffused in the air. Our assumption was verified in a study by Kim et al. [19], in which the PSD during the process of pouring silica NPs (7 nm) into a container measured a mode diameter of 100 nm, higher than the background concentration.

The average PNC in the workplace was approximately 30,000 pt/cc. This value was similar to that in workplaces producing powders such as nano TiO_2_ and silver [20], nano-silica [21] and lower than in workplaces where NPs are generated by heat such as welding or rubber fume [22].

In this study, the worker’s average LDSA concentration was 51 mm^2^/cm^3^. This value was similar to the results of a study by Ham et al. [23], which showed that the average LDSA in a workplace manufacturing engineered NPs ranged between 37 and 93 mm^2^/cm^3^.

The patient in this study (Worker A) contracted an ILD. However, in the previous three PAP cases, the ITO target manufacturing processes were milling (US), polishing (US), or sandblasting (China). The common feature of these three processes is that a physical impact is applied to the ITO target, and these physical impact and high heat processes are known as NPs generation processes. Therefore, variations were expected in the degree of NPs generation depending on the specific ITO industry process. Therefore, it was necessary to investigate the concentration levels of occupational exposure to indium NPs for the various processes.

### 4.2. Occupational Exposure Limit (OEL) for Indium Nanoparticles

The ACGIH has only a standard threshold limit value defined for the total indium and does not regard the particle size. However, they have recently introduced a respirable fraction (50% cut-point 4 *µ*m) regarding the particle size that can reach deep into the lungs when inhaled. It is currently the lowest indium OEL globally. However, this fraction does not typically consider NPs. According to the ICRP model, the 4-μm-sized particles’ deposition rate in the alveolar region is as low as 5% (50% cut-point at 30 nm). Therefore, it is appropriate to set the OEL based on the nanoscale for particles with nanotoxicity.

In this study, the fraction of indium NPs in respirable indium dust was found to be only 2.7% in the ITO powder handling process, demonstrating that the occupational exposure assessment cannot be sufficiently performed by monitoring the respirable dust alone. Furthermore, since process-dependent differences in the indium NPs fraction in respiratory dust are expected in the ITO industry, it would be appropriate to separately monitor the concentration of indium NPs and set the OEL accordingly.

Currently, separate exposure standards for NPs are set only for some nanomaterials. For example, TiO_2_ has a respirable dust size of 3 mg/m^3^ and a NP size of 0.1 mg/m^3^. Moreover, 20,000 and 40,000 particles/cm^3^ are the threshold values set for metal oxides with biopersistence depending on density (6000 kg/m^3^) [24].

Therefore, the OEL for indium NPs should be less than 0.0001 mg/m^3^, which is the respirable TLV, or less than approximately 3000 particles/cm^3^ (the number of particles estimated by conversion when a 20 nm indium particle equals 0.0001 mg/m^3^).

### 4.3. Indium-Related Lung Diseases

Indium is predominately obtained as a by-product in the process of zinc smelting. Currently, indium is primarily used to produce ITO, a raw material for making the apparatus for properly displaying the mark of a display device, such as the plant where Worker A worked. In 2001, a 27-year-old Japanese male worker engaged in an ITO target polishing process contracted bilateral pneumothorax secondary disease. After this case was reported in 2003 [25], a report on the clinical features of 10 indium-related ILD cases (two in the US, one in China, and seven in Japan) was released in 2010 [1]. Based on radiologic features, the authors reported that three out of these 10 patients showed clinical features of PAP, six clearly showed fibrotic changes, of whom five patients had confirmed cases of traction bronchiectasis or bronchiolectasis, and honeycombing was observed in three cases.

This seminal report was followed by a five-year longitudinal study (2008–2011) in Japan, in which 240 workers exposed to indium and 40 workers enrolled as controls were monitored for five years. According to the report, no significant differences were observed in the progression rate of interstitial lesions between the exposure group (10 out of 172 subjects) that was administered HRCT, and the control group (two out of 35 subjects), with only a statistically significant difference in the rate of progression of emphysematous changes [26]. Only the association with emphysematous changes is reported in other studies [27].

Moreover, a previous study conducted in Korea, presumably including worker A from this study, observed no association between the ILD clinical features and blood indium concentration from an HRCT analysis of 34 patients [28]. However, a comparison of the blood indium concentration in two groups with and without interstitial lesions using HRCT scan images revealed a significantly higher blood indium concentration in the group with interstitial lesions (*n* = 12) than in the group without interstitial lesions (*n* = 38) [8].

Pulmonary disease caused by indium exposure has been investigated and reported by experts in various fields since the first report on a very young patient was released in 2001. However, only a limited number of studies have been conducted. A comprehensive study was conducted in 2012 analyzing 10 patients reported that indium induced ILD showed different clinical features, such as fibrosis and PAP. Thus, it is challenging to determine a specific type of ILD associated with indium exposure. Moreover, several studies failed to present statistically significant findings regarding ILD incidence and exacerbation. However, many studies have confirmed that the serum indium concentration and the concentration of the Krebs von den Lungen-6 (KL-6), an ILD biomarker, are associated with indium-induced ILD, which serves as the basis for assuming that indium exposure leads to ILD.

The studied patient was directly exposed to IO and ITO dust in the ITO manufacturing process from 2007, during procedures like manually pouring the IO and ITO mixture into the mixing container and exchanging the full container collecting the ITO powder.

Overall, the indium exposure levels measured three times over a four-month period (January–April 2019) in this study to assess the workplace environment were not very high. However, an epidemiological survey conducted by the Occupational Safety and Health Research Institute (OSHRI) in Korea on several domestic workplaces handling indium from 2012 to 2013, including Worker A’s workplace. Since then, engineering improvements have been implemented to reduce the diffusion of indium dust in workplaces (i.e., blocking dust diffusion under the spray dryer, applying a partition to prevent dust diffusion in the upper part of the dryer, installing dust collectors, scrubbers). For this reason, it is not appropriate to estimate the past indium concentrations to which the patient was exposed based on the workplace environment evaluation conducted in 2019. Moreover, according to the results of the OSHRI survey in 2012, the indium concentration (*n* = 9) in the total dust in the Korean indium target manufacturing industry produced an arithmetic mean of 121 µg/m^3^, a geometric mean of 54.7 µg/m^3^, and a maximum value of 502.1. The concentrations measured in this study were 27.3 µg/m^3^, 18.0 µg/m^3^, and 68.4 µg/m^3^, respectively, which confirms that the past concentration levels [14].

Meanwhile, indium was not detected in the two control group serum samples. However, the levels detected in Worker A and the onsite manager (working period: 2012–2019) were 43 and 34 µg/L, respectively, significantly exceeding the Japanese OEL for the indium concentration in workers’ serum (3 μg/L) [29]. The higher indium concentration for Worker A, who stopped working 16 months prior to the study, indicates that the indium concentrations to which Worker A was exposed in the past was higher than those measured in this study.

According to a Japanese study, the mean serum indium concentration of workers exposed to indium dust in the past was 9.63 mg/L (range: ND~126.8, *n* = 127) [4]. According to other past studies in Korea, the GM ±GSD serum indium concentration in subjects with interstitial changes on HRCT were 16.3 ± 4.3 mg/L (*n* = 12), which were significantly higher than the GM ± GSD in subjects without interstitial changes on HRCT (3.3 ± 12.5 mg/L, *n* = 38) [8]. Compared with these previously published results, it was confirmed that Worker A was exposed to high concentrations of indium.

To summarize, Worker A was exposed to indium for approximately five years when abnormal X-ray findings detected pulmonary defects in 2012 while participating in an epidemiological study. Because the serum indium concentration of Worker A, who is no longer exposed to indium, exceeded that of the current worker, we established that the indium exposure level was higher in the past compared to the present level. Moreover, Worker A’s CT findings indicate defects similar to those caused by interstitial pneumonia/pulmonary fibrosis associated with indium-induced lung diseases. Therefore, we concluded that Worker A’s condition was an occupational indium lung disease case.

## 5. Conclusions

Recent studies have shown that indium NPs have a higher pulmonary toxicity than other metal NPs, and long-term exposure to indium can cause lung diseases such as PAP and pulmonary fibrosis. In this study, the occupational exposure level to indium NPs in an ITO powder manufacturing plant was investigated for the first time. The concentrations of indium NPs measured using NRD samplers were lower than the ACGIH TLV for respirable indium (0.0001 mg/m^3^) for all the samples. However, the mean fractions of indium NPs in the total and respirable dust were as low as 0.4% and 2.7%, respectively, suggesting that the exposure assessment for respirable indium alone is not sufficient to adequately understand the occupational exposure to indium NPs. Therefore, it is necessary to conduct research on indium NPs exposure in various processes generating ITO dust to further investigate the effects of indium on workers and facilitate the necessary implementation of indium-reducing technologies. It is also essential to set separate OEL values for indium NPs.

## Figures and Tables

**Table 1:** Indium concentrations and fraction (%) of respirable dust and nanoparticles (NPs) in workers’ personal samples.

		Wet Ball Mill	Indium Concentration (mg/cm^3^)			Fraction (%)	
Workers			in TD ^3^	in RD ^4^	in NPs ^5^	RD/TD	NPs/TD
Manufacturing worker	1st day	No	0.0402	NA ^6^	0.008 × 10^−2^	NA	0.2
	2nd day	Yes	0.0684	NA	0.010 × 10^−2^	NA	0.1
	3rd day	No	0.0136	0.093 × 10^−2^	NA	6.8	NA
Managing workers A ^1^		No	0.0085	NA	0.003 × 10^−2^	NA	0.4
Managing workers B ^2^		No	0.0060	0.166 × 10^−2^	NA	27.7	NA

^1^ ManagingWorker A who multitasks (manufacturing and management) in the indium tin oxide (ITO) processes.; ^2^ managing Worker B who multitasks (manufacturing and management) in indium oxide (IO) and ITO processes.; ^3^ total dust; ^4^ respirable dust; ^5^ nanoparticles.; ^6^ not available.

**Table 2:** Indium concentrations and fraction (%) of RD and NPs in areasamples.

Location	Sampling Date	Wet Mill	Dust (mg/cm^3^)		Indium Concentration(mg/cm^3^)			Fraction (%)		
			TD ^1^	RD ^2^	in TD	in RD	in NPs ^3^	RD/TD	NPs/RD	NPs/TD
Wet Mill	t	No	0.0434	0.0193	0.050 × 10^−2^	NA	ND ^4^	NA	NA	NA
	2nd	Yes	NA	NA	1.770 × 10^−2^	NA	ND	NA	NA	NA
	3rd	No	NA	NA	0.010 × 10^−2^	0.007 × 10^−2^	ND	70	NA	NA
Mixer	1st	No	0.0632	0.0241	0.470 × 10^−2^	NA	ND	NA	NA	NA
	2nd	No	NA	NA	1.010 × 10^−2^	0.228 × 10^−2^	0.003 × 10^−2^	22.6	1.3	0.3
Vibration classifier	1st	No	0.0581	0.0256	0.360 × 10^−2^	NA	ND	NA	NA	NA
	2nd	No	NA	NA	0.870 × 10^−2^	0.095 × 10^−2^	0.004 × 10^−2^	10.9	4.2	0.5
PC table in corridor	1st	No	0.0853	0.0198	NA	NA	NA	NA	NA	NA
	2nd	No	NA	NA	0.160 × 10^−2^	0.037 × 10^−2^	0.001 × 10^−2^	23.1	2.7	0.6
End of corridor		NA ^4^	NA	NA	0.080 × 10^−2^	0.019 × 10^−2^	NA	23.8	NA	NA

^1^ Total dust; ^2^ respirable dust; ^3^ nanoparticles; ^4^ not available.

**Table 3:** Particle number concentration, and diameter using Scanning Mobility Particle Sizer, and Optical Particle Sizer by sampling positions.

Date	Measurement Points	SMPS ^3^			OPS ^4^	
		GM ^5^ of NC ^6^ (GSD ^7^) (range)	Group ^8^	Particle Diameter ^9^	GM of NC ^1^ (GSD) (range)	Group
1st day	Workshop ^1^	34,629 (1.12) (26,144–43,170)	3	39	1000 (1.13) (758–1367)	2
	Corridor	45,890 (1.20) (37,553–62,275)	2	37	1140 (1.08) (1021–1376)	1
	Outdoor	51,907 (1.76) (22,236–201,191)	1	53	1199 (1.06) (1077–1350)	1
3rd day	Workshop ^2^	23,148 (1.27) (13,819–38,952)	2	82	5074 (1.27) (3005–7558)	1
	Corridor	20,117 (1.06) (18,589–22,659)	3	85	3602 (1.02) (3464–3702)	2
	Outdoor	35,351 (1.16) (25,848–45,808)	1	59	4053 (1.08) (3558–4486)	2

^1^ Center of the workshop; ^2^ in front of vibration classifier; ^3^ scanning mobility particle sizer; ^4^ optical particle sizer; ^5^ geometric mean; ^6^ particle number concentration (unit: particles/cm^3^); ^7^ geometric standard deviation; ^8^ grouping codes from Scheffe’s multiple range tests for multiple comparisons. Significant difference between groups at *p* < 0.01; ^9^ mode diameter (unit: nm).

## Data Availability

Not applicable.
